# Colorectal neoplasia is not uncommon in siblings aged under 50 years of patients with early-onset colorectal advanced adenomas: A cross-sectional study

**DOI:** 10.1097/MD.0000000000043222

**Published:** 2025-07-04

**Authors:** Luan Minh Dang, Nhan Quang Le, Huy Minh Le, Diem Thi-Ngoc Vo, Nguyen Lam Vuong, Minh Cuong Duong, Duc Trong Quach

**Affiliations:** a Department of Internal Medicine, School of Medicine, University of Medicine and Pharmacy at Ho Chi Minh City, Ho Chi Minh City, Vietnam; b Department of Gastroenterology, University Medical Center, Ho Chi Minh City, Vietnam; c GI Endoscopy Department, University Medical Center, Ho Chi Minh City, Vietnam; d Department of Histology-Embryology and Pathology, School of Medicine, University of Medicine and Pharmacy at Ho Chi Minh City, Ho Chi Minh City, Vietnam; e Department of Medical Statistics and Informatics, Faculty of Public Health, University of Medicine and Pharmacy at Ho Chi Minh City, Ho Chi Minh City, Vietnam; f School of Population Health, University of New South Wales, Sydney, NSW, Australia.

**Keywords:** adenoma, advanced adenoma, colorectal neoplasms, early-onset colorectal cancer, screening, siblings

## Abstract

Research on the prevalence of adenomas and colorectal cancer (CRC) in individuals aged 50 years or younger with first-degree relatives diagnosed with early-onset colorectal advanced adenoma (E-CAA), defined as the onset of colorectal advanced adenoma (CAA) at or before 50 years of age, is scarce. We examined the prevalence of colorectal neoplasia among siblings aged 50 years or younger of E-CAA patients. A cross-sectional study was conducted at the University Medical Center in Ho Chi Minh City, Vietnam. Study participants included 96 siblings aged 50 years or younger from 58 E-CAA patients, who were selected from a total of 732 identified siblings, with a notably high rate of participation refusal. All included siblings were either older than the patients diagnosed with E-CAA or younger by no more than10 years. Individuals with a family history of CRC were excluded from this study. All siblings underwent standard bowel preparation and colonoscopies. The mean age of siblings was 40.9 ± 6.0 years, with males comprising 47.9%. Most of the siblings (83.3%) were asymptomatic. The prevalence of colorectal neoplasia among the siblings was 25%. Of these, 95.8% were adenomas, and CRC was identified in one sibling. The proportion of CAA and advanced colorectal neoplasia was 6.3% and 7.3%, respectively. These proportions increased to 13% and 15.2%, respectively, among the male siblings. None of the female siblings were diagnosed with CAA or advanced colorectal neoplasia. No statistically significant difference was observed in the prevalence of colorectal neoplasia (23.7% vs 25.9%, *P* = .81), CAA (5.3% vs 6.9%, *P* > .99), or advanced colorectal neoplasia (7.9% vs 6.9%, *P* > .99) between sibling age groups (40 years or 40–50 years). Colorectal neoplasia is not uncommon in siblings aged ≤ 50 years of E-CAA patients. The prevalence of CAA and advanced colorectal neoplasia is markedly elevated in male siblings. These high-risk individuals may benefit from early targeted colorectal cancer screening.

## 1. Introduction

Globally, colorectal cancer (CRC) ranks as the third most prevalent malignancy and the second most frequent cause of cancer mortality.^[1]^ Colorectal adenomas are considered significant precursors of CRC.^[2,3]^ Colorectal advanced adenomas (CAA), defined as adenomas exhibiting at least one of the following features: size ≥ 1 cm (i.e., large adenomas), a villous component ≥ 25%, or high-grade dysplasia, are associated with an increased risk of CRC development.^[4,5]^ Screening and removal of adenomas and CAA play a critical role in lowering both the incidence and mortality rates of CRC.

Traditionally, CRC screening is advised to begin at age 50 in average-risk individuals.^[2,3]^ However, the rising incidence of early-onset CRC, which is defined as CRC occurring in individuals aged 50 years or younger, has prompted a reevaluation of these guidelines.^[6,7]^ Recent United States guidelines have recommended lowering the age of CRC screening initiation from 50 to 45 years.^[8,9]^ In contrast, the third Asia-Pacific consensus on CRC screening continues to recommend starting screening at age 50 for average-risk individuals due to limited cost-effectiveness data supporting earlier screening in the region.^[10]^ As a result, CRC screening is typically not advised for those aged 45 to 50 years. Implementing routine screening in this population could impose substantial challenges related to rising healthcare costs and workload burden. Therefore, a more practical approach is risk-based stratification and prioritization of high-risk individuals for early screening.

Having a first-degree relative (FDR) diagnosed with CRC doubles an individual’s risk of developing the disease.^[11]^ Moreover, the risk of developing CRC increases to over 3-fold when the affected FDR is 50 years of age or younger.^[11,12]^ Individuals under 50 years of age with a FDR diagnosed with CRC also exhibited a 3.17 times greater likelihood of disease development.^[13]^ Although the association between CRC development and family history of CRC has been extensively studied, limited data are available on the risk in individuals with FDRs diagnosed with CAA. Studies assessing the association between CAA and CRC risk in individuals with a family history of CAA in FDRs have typically focused on individuals aged ≥ 40 years.^[14–16]^ Additionally, there is inconsistency in how CAA is defined in the available studies. Some studies exclusively investigated the size, while others only examined the villous components of adenomas.^[14,15,17]^ Further research is warranted to better elucidate the prevalence of CAA and CRC in individuals aged ≤ 50 years with FDRs diagnosed with early-onset CAA (E-CAA), defined as a diagnosis at or before the age of 50. Our prior case-control study demonstrated a heightened risk of CAAs in siblings aged 50 or younger of patients with E-CAAs.^[18]^ The present study examined the prevalence and characteristics of colorectal adenomas, CAA, and CRC in Vietnamese individuals aged ≤ 50 years old with FDRs diagnosed with E-CAA. It provides prevalence estimates for this high-risk population, addressing a critical gap in understanding the disease burden. Additionally, it offers essential information for clinical decision-making in CRC screening, particularly in Vietnam and comparable Asian countries where screening resources are constrained.

## 2. Methods

### 2.1. Study design and selection of study participants

This cross-sectional study was carried out at the GI Endoscopy Department, University Medical Center (UMC) in Ho Chi Minh City, Vietnam, from September 1st, 2021 to September 30th, 2024. Ethical approval for this study was granted by the Ethics Committee of the University of Medicine and Pharmacy in Ho Chi Minh City, Vietnam (reference No. 345/HDDD-DHYD). Written informed consent was obtained from all participants or their legal representatives prior to their inclusion in the study.

During the study period, all individuals between 18 and 50 years of age with a confirmed diagnosis of CAA through colonoscopic and histological evaluation at the UMC were approached and invited to enroll in the study and were referred to as probands. The exclusion criteria for the probands included a personal or family history of CRC, the presence of hereditary CRC syndromes (e.g., Lynch syndrome, familial adenomatous polyposis, Peutz-Jeghers syndrome, and juvenile polyposis), or inflammatory bowel disease. Data were collected from probands who consented to participate and included the number of siblings, their contact details, and causes of death in cases where the siblings had died. Young siblings, defined as those between 18 and 50 years of age, were contacted either in person or by phone and invited to participate in this study. The exclusion criteria included the presence of CRC alarming features (e.g., hematochezia, unexplained weight loss, iron deficiency anemia, or a positive fecal occult blood test), a personal or family history of CRC, known hereditary CRC syndromes, inflammatory bowel disease, previously diagnosed colonic adenomas or CRC, a history of colonic surgery, colonoscopy performed in the previous 5 years, medical contraindications to colonoscopy, or logistical barriers preventing the procedure from being performed at the study site, such as residing abroad. Current guidelines advise starting CRC screening in individuals with an FDR diagnosed with CAA at the age of 40 or 10 years before the earliest diagnosis of CAA in FDRs, whichever is earlier.^[8,19]^ Therefore, eligibility for siblings was restricted to individuals who were either older than their probands or younger by no more than 10 years. Following counseling on adenoma, CAA, and CRC risks, these siblings were invited to undergo a colonoscopic evaluation.

### 2.2. Sample size calculation

The minimum number of participants required for the study was calculated using the standard formula for sample size in a cross-sectional study, with an absolute error of 5% and a type 1 error of 5%.^[20]^ According to a previous study, the prevalence of large adenomas in individuals with FDRs diagnosed with large adenomas was 5.4%.^[14]^ The minimum required sample size was estimated to be 79. Considering a 20% non‐response rate, at least 95 participants were recruited to meet the required sample size for the statistical analysis.

### 2.3. Colonoscopy procedure and histological evaluation

Pre-colonoscopy bowel preparation was performed using 3 liters of polyethylene glycol-based solution (Fortrans^®^, Ipsen Industrie, France). All colonoscopies were performed in accordance with UMC-established protocols and carried out by qualified endoscopists who had an adenoma detection rate of at least 30%. All colonoscopic procedures were performed under conscious sedation, administered via intravenous propofol, utilizing standard high-definition colonoscopes (Olympus Evis Exera III High Definition CV-190; Olympus Co., Ltd., Tokyo, Japan). Bowel preparation quality was assessed during the procedure. Study participants were excluded if they had inadequate bowel preparation, defined as a total Boston Bowel Preparation Scale (BBPS) score of <6 or a score of <2 in any of the 3 sections.^[21]^ Additionally, individuals in whom cecal intubation was not achieved, as confirmed by visualization of the appendiceal orifice and ileocecal valve, were excluded. The withdrawal times were maintained at a minimum of 6 minutes to optimize adenoma detection.^[22]^ Detected polyps were assessed with respect to size, morphological characteristics, and anatomical site. The size of polyps was assessed using open biopsy forceps measuring 7 mm in width or open polypectomy snares with a diameter of 10 mm. The Paris classification system was used to assess the polyp morphology.^[23]^ The anatomical location was classified into 2 groups: the proximal colon (i.e., the cecum, ascending colon, hepatic flexure, and transverse colon) and the distal colon (i.e., the splenic flexure, descending colon, sigmoid colon, and rectum). All polyps detected during the procedure were excised and subjected to histopathological examination.

Resected polyps and biopsy samples were fixed in 10% buffered formalin, processed using standard UMC protocols, and examined histologically by 2 certified gastrointestinal pathologists following the World Health Organization criteria.^[24]^ Any disagreements between the 2 pathologists were discussed until consensus was reached.

### 2.4. Data collection

Participant data, encompassing demographic details (age and sex), indications for colonoscopy, colonoscopic findings (including the location, size, quantity, and morphology of polyps and colorectal cancer), histological findings, and additional relevant information, were systematically recorded using a standardized data collection form. Indications for colonoscopy were grouped into 4 categories: (1) CRC screening; (2) abdominal pain; (3) changes in bowel habits; and (4) miscellaneous indications, including examinations of prolapsed hemorrhoids, anal fissures, or various anorectal disorders. Histopathological findings encompassed adenoma subtypes (tubular, tubulovillous, villous) alongside other polyp types (sessile serrated lesions, traditional serrated adenomas, or hyperplastic polyps) as well as the degree of dysplasia (low- or high-grade). Other collected data included body mass index, smoking status, regular nonsteroidal anti-inflammatory drug and aspirin use, alcohol consumption, and BBPS score. Smoking status was classified into current smoker, ex-smoker, or never smoker categories. Regular aspirin use was defined as a minimum daily dose of 75 mg, while nonsteroidal anti-inflammatory drug use was considered regular if taken during the past month. Alcohol consumption was defined as a regular intake of 20 grams or more per day.

### 2.5. Outcomes

The primary outcome was the prevalence of CAA and CRC in young siblings of E-CAA patients. Secondary outcomes were the prevalence of all colorectal adenomas and advanced colorectal neoplasia (ACN) in the siblings. In this study, all colorectal lesions were identified by endoscopy and histology. CAA was characterized by any of the following features: adenoma size ≥ 10 mm, a villous component of at least 25%, high-grade dysplasia, or a combination of these criteria.^[4,5]^ CRC was defined as histopathologically confirmed adenocarcinoma. ACN included CAA and CRC, while colorectal neoplasia comprised adenomas and CRC. Multiple adenomas were defined as the presence of at least 3 adenomas.

### 2.6. Statistical analysis

Data were analyzed using R software version 4.1.3 (R Core Team, Vienna, Austria, 2020). Categorical variables, presented as absolute counts and percentages, were analyzed using Chi-squared or Fisher exact tests as applicable. For comparison, 95% confidence intervals (95% CIs) of the point estimates for the prevalence of colorectal neoplasia, adenoma, advanced adenoma, and advanced colorectal neoplasia were calculated. Normally distributed continuous variables were presented as mean ± standard deviation. Alpha was set at the 5% level.

## 3. Results

### 3.1. Baseline characteristics of study participants

**Figure 1.**
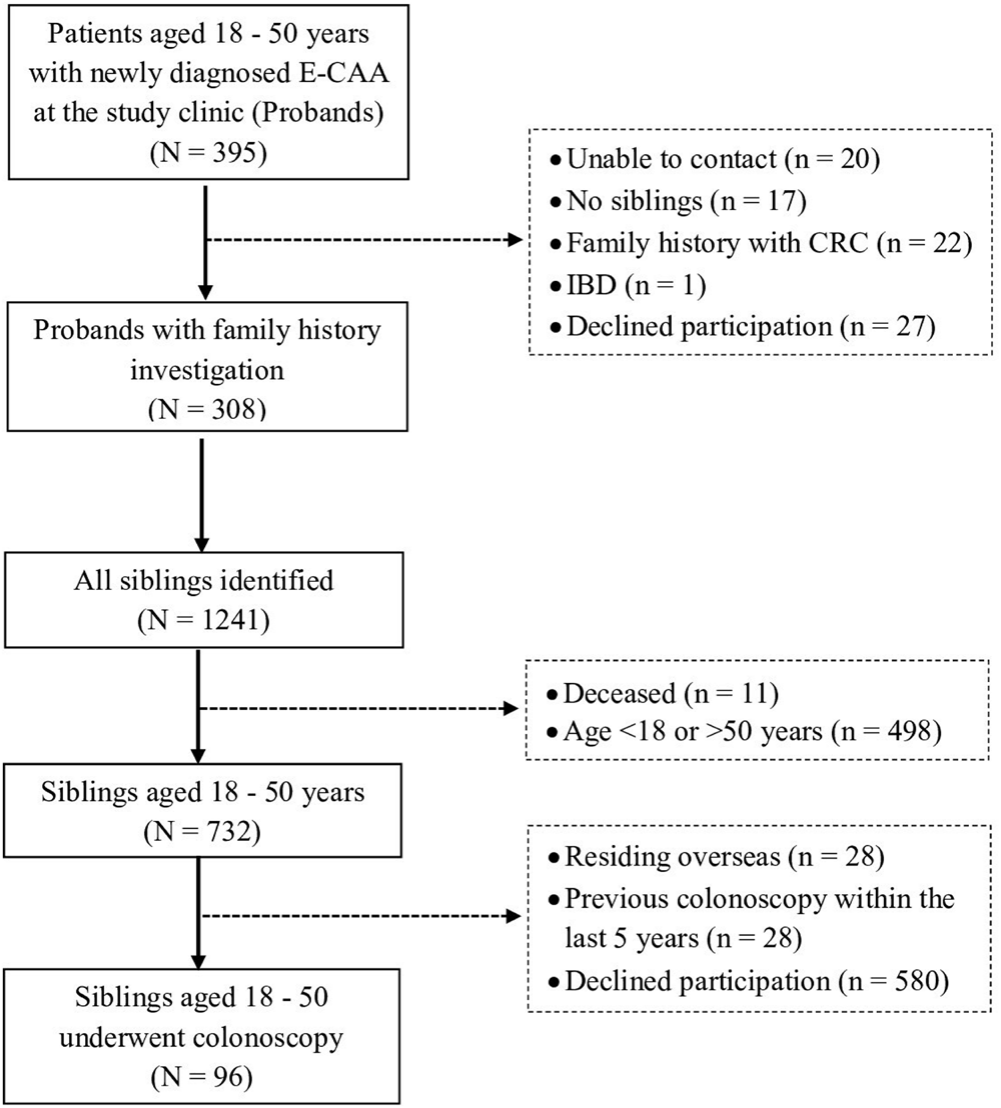
Flowchart of study participants. CRC = colorectal cancer; E-CAA = early colorectal advanced adenoma; IBD = inflammatory bowel disease.

During the study period, 395 probands who were diagnosed with E-CAAs at the UMC were identified (Fig. 1). Among these probands, 308 met the inclusion criteria and provided information on 1241 siblings. Of these siblings, 11 were deceased and 498 were outside the eligible age range. Of the remaining 732 siblings aged between 18 and 50 years old, 96 siblings from 58 probands fulfilled the inclusion criteria and were enrolled in the study.

Among 58 probands, the mean age was 42.1 ± 6.3 years, and 53.4% were males. CAA was recorded in the proximal colon, distal colon, and synchronous adenomas in 19%, 77.6%, and 3.6%, respectively (data not shown).

The 96 recruited young siblings had a mean age of 40.9 ± 6.0 years, with males representing 47.9%, and never smokers constituting 85.4% (Table 1). Most (83.3%) were asymptomatic, undergoing colonoscopy for CRC screening. All participants attained BBPS scores ≥ 7. Complete cecal intubation was achieved in all colonoscopies without any reported procedural complications (data not shown).

**Table 1:** Baseline characteristics of study participants (n = 96).

Characteristics*	
Age (years)	40.9 ± 6.0
<40	38 (39.6)
40–50	58 (60.4)
Male	46 (47.9)
BMI (kg/m^2^)	23.2 ± 2.9
Smoking status	
Never smoked	82 (85.4)
Ex-smoker	4 (4.20)
Current smoker	10 (10.4)
Aspirin use†	0
NSAIDs use‡	3 (3.1)
Alcohol intake§	13 (13.5)
Indications for colonoscopy	
CRC screening	80 (83.3)
Abdominal pain	4 (4.2)
Changes in bowel habits	9 (9.4)
Others∥	3 (3.1)
BBPS score	
7	7 (7.3)
8	11 (11.5)
9	78 (81.2)

BBPS = Boston Bowel Preparation Scale, BMI = body mass index, CRC = colorectal cancer, NSAID = nonsteroidal anti-inflammatory drug.

* Categorical variables are presented as No. (%), and continuous variables are presented as mean ± standard deviation.

† Aspirin use: regular use of aspirin ≥ 75 mg per day during the previous month.

‡ NSAIDs use: regular use of NSAIDs during the previous month.

§ Alcohol intake: alcohol consumption ≥ 20 g per day.

∥ Hemorrhoids, bloating, and tenesmus.

### 3.2. Proportions of colorectal neoplasia among siblings of patients diagnosed with early-onset colorectal advanced adenomas

All colorectal neoplasias were observed in 25% (24/96, 95% CI 17.4–34.5%) of siblings (Table 2). The proportion of adenomas was 24% (23/96, 95% CI 16.5–33.4%), and that of CRC was 1% (1/96). The sibling with CRC had no alarming features, underwent colonoscopy due to abdominal pain, and exhibited a cancerous lesion that greatly narrowed the bowel lumen, preventing the completion of colonoscopy. The sibling’s proband exhibited an adenoma with high-grade dysplasia (data not shown).

**Table 2:** Proportions of colorectal neoplasia among siblings of patients diagnosed with early-onset colorectal advanced adenomas (n = 96).

Colorectal neoplasia	No. (%)
All colorectal neoplasia	24 (25.0)
Adenomas	23 (24.0)
Proximal adenomas*	6 (6.3)
Distal adenomas†	14 (14.6)
Synchronous adenomas‡	3 (3.1)
Multiple adenomas§	5 (5.2)
Advanced adenoma∥	6 (6.3)
Adenomas ≥ 10 mm	6 (6.3)
Tubulovillous or villous	0
High-grade dysplasia	0
Colorectal cancer	1 (1)
Advanced colorectal neoplasia¶	7 (7.3)

* Proximal adenomas^:^ proximal to the splenic flexure.

† Distal adenomas: distal to the splenic flexure.

‡ Synchronous adenomas: adenomas detected in both proximal and distal colons.

§ Multiple adenomas: ≥ 3 adenomas.

∥ Advanced adenoma: adenomas ≥ 1 cm, adenomas with villous elements ≥ 25% or high-grade dysplasia.

¶ Advanced colorectal neoplasia: advanced adenoma or colorectal cancer.

Among all siblings, distal and proximal adenomas accounted for 14.6% (14/96) and 6.3% (6/96) of cases, respectively. A total of 3.1% (3/96) of siblings exhibited adenomas detected in both the proximal and distal colons. Siblings with 3 or more adenomas accounted for 5.2% (5/96) of cases.

The proportion of CAA was 6.3% (6/96, 95% CI 2.9–13.0), while that of ACN was 7.3% (7/96, 95% CI 3.6–14.3). All siblings with CAA were diagnosed based on the size of the adenomas, which was ≥10 mm. Tubulovillous, villous adenomas, and adenomas with high-grade dysplasia were not detected in any sibling.

### 3.3. Distribution of colorectal neoplasia among siblings by age and gender

Among siblings aged < 40 years, the proportion of all colorectal neoplasias was 23.7% (9/38), with 8 siblings (21.1%) diagnosed with adenomas, 2 (5.3%) with CAA, and 3 (7.9%) with ACN (Table 3). Among siblings aged between 40 and 50 years old, the proportion of all colorectal neoplasias was 25.9%, and all lesions were adenomas. CAA and ACN were observed in 6.9% (4/58) of cases. No statistically significant differences were observed between the age groups regarding the proportion of adenomas (*P* = .59), CAA (*P* > .99), ACN (*P* > .99), or all colorectal neoplasia (*P* = .81).

**Table 3:** Distribution of colorectal neoplasia among siblings by age and gender (n = 96).

Colorectal neoplasia*	Age group (years)			Gender		
	<40 (n = 38)	40–50 (n = 58)	*P* value†	Male (n = 46)	Female (n = 50)	*P* value†
All colorectal neoplasia‡	9 (23.7)	15 (25.9)	.81	13 (28.3)	11 (22)	.48
Adenomas	8 (21.1)	15 (25.9)	.59	12 (26.1)	11 (22)	.64
Advanced adenomas§	2 (5.3)	4 (6.9)	>.99	6 (13)	0	**.01**
Colorectal cancer	1 (2.6)	0	.40	1 (2.2)	0	.48
Advanced colorectal neoplasia∥	3 (7.9)	4 (6.9)	>.99	7 (15.2)	0	**.004**

Bold values: *P* < .05.

* Data are presented as No. (%).

† Fisher exact test.

‡ All colorectal neoplasia: adenomas or colorectal cancer.

§ Advanced adenoma: adenomas ≥1 cm, adenomas with villous elements ≥ 25% or high-grade dysplasia.

∥ Advanced colorectal neoplasia: advanced adenoma or colorectal cancer.

Among the male siblings, the proportion of all colorectal neoplasias was 28.3% (13/46), with 12 siblings (26.1%) diagnosed with adenomas, 6 (13%) with CAA, and 7 (15.2%) with ACN. Among the female siblings, the proportion of all colorectal neoplasia cases was 22%, and all were diagnosed with adenomas. The proportion of adenomas (*P* = .64) or all colorectal neoplasia (*P* = .48) did not differ significantly between male and female siblings. Significantly higher rates of CAA (*P* = .01) and ACN (*P* = .004) were observed in male siblings compared to their female counterparts.

## 4. Discussion

This study revealed that colorectal neoplasia and ACN were not uncommon in individuals ≤ 50 years, especially in males, who have siblings diagnosed with E-CAA. Adenomas and CAA were the most common lesions in these siblings.

We found that the prevalence of all colorectal neoplasias among siblings ≤ 50 years of E-CAA patients was 25% (95% CI 17.4–34.5%). The observed prevalence exceeded the the rate of 13.7% (95% CI 11.2–16.8%) reported for average-risk individuals in a recent review.^[25]^ Among the siblings diagnosed with colorectal neoplasia in our study, 95.8% (23/24) had colorectal adenomas, whereas none were diagnosed with serrated polyps, including sessile serrated lesions and traditional serrated adenomas. A recent meta-analysis revealed that the prevalence of sessile serrated lesions in Asian countries was low (2.6%), with an increase in the number of observed cases with advancing age.^[26]^ Another study conducted in Vietnam showed that the prevalence of sessile serrated lesions in individuals aged ≤ 50 years was 2.9% (23/780).^[27]^ Traditional serrated adenomas are also uncommon, constituting <1% of all colorectal polyps.^[28]^ The relatively small sample size of our study and the exclusive recruitment of participants aged ≤ 50 years may partially explain the low prevalence of serrated polyps observed. Additionally, sessile serrated lesions are generally characterized by a flat or sessile morphology, often accompanied by a mucous cap on the surface. The presence of these features presents considerable obstacles to the accurate identification of lesions during colonoscopy.^[26,27]^ Indeed, a discrepancy in the endoscopists’ ability to detect sessile serrated lesions has been reported.^[29]^ Other studies conducted in Asian countries indicated that adenomas are more prevalent than serrated polyps in individuals aged 50 years or younger.^[30,31]^ Previous studies have also shown that adenomas represent the predominant neoplastic lesions observed in the FDRs of patients diagnosed with CAA.^[16]^ CRC was uncommon in our study, with only one sibling detected with this condition. Although early-onset CRC is on the rise,^[6,7]^ its prevalence among individuals younger than 50 years of age remains low, with earlier studies estimating rates between 0.1% and 0.9%.^[30,32–34]^

We found that the prevalence of all adenomas in siblings aged 50 years or younger of E-CAA patients was 24% (95% CI 16.5–33.4%), surpassing the prevalence reported in previous studies, which ranged from 7.3% (95% CI 6.8–7.8) to 12.1% (95% CI 11.3–13).^[31,32,35,36]^ A previous study found that siblings of patients with colorectal CAA had an increased risk of adenoma development compared to those without a family history.^[16]^ In our study, CAA was found in 6.3% (95% CI 2.9–13.0) of siblings aged ≤ 50 years of patients diagnosed with E-CAA. The observed prevalence exceeded that of CAA among individuals aged 50 years or younger, which ranged from 1.05% (95% CI 0.5–2.3) to 1.6% (95% CI 1.4–1.9) in previous studies.^[30,34,35]^ It has been documented that FDRs of CAA patients had a higher risk of developing the same lesions in comparison to those without such a family history.^[15,16]^ In our study, we found that colorectal adenomas and colorectal advanced neoplasia were not uncommon in siblings ≤ 50 years of E-CAA patients. The reasons for this phenomenon are not fully understood but may involve genetic predisposition and shared environmental factors, including diet, lifestyle, and smoking. The interaction between these genetic and nongenetic factors may contribute to adenoma development and progression in these individuals.^[16,37–39]^

CRC screening is advised to begin between ages 45 and 50 for individuals at average risk, according to current guidelines.^[8,10,19]^ Consequently, CRC screening is not typically implemented in individuals under the age of 45 to 50 years. Routine screening in this population may lead to an escalation in costs and the workload burden of healthcare systems, especially in Asian countries with limited resources. Implementing CRC risk stratification and prioritizing screening in high-risk populations offer more pragmatic solutions. Our previous case-control study reported an odds ratio of 6.33 for CAAs in siblings of E-CAA patients compared to controls and supported the endorsement of early CRC screening in this group.^[18]^ In the present study, we found a prevalence of 25% for colorectal neoplasia, 6.3% for CAAs, and 7.3% for ACN, highlighting the substantial disease burden in this high-risk group. In addition, all siblings recruited in this cross-sectional study were either older than their probands with E-CAAs or younger by no more than 10 years. The ages of these siblings were aligned with the recommendations established in the current guidelines.^[8,19,40]^ In light of our findings demonstrating a higher prevalence of adenomas and CAA among these siblings, it is crucial to stratify CRC risk in individuals under 50 years by incorporating a family history of FDRs with CAA. Our results also reinforce the recommendation for early CRC screening in this high-risk group, aligning with current guidelines for FDRs of CAA patients.

We observed no significant variation in adenoma and CAA prevalence between the sibling age groups. Among siblings aged ≤ 40 years, the prevalence of colorectal neoplasia and ACN was 23.7% (95% CI 13–39.2) and 7.9% (95% CI 2.7–20.8), respectively. The observed prevalence of all colorectal neoplasia exceeded the prevalence in average-risk individuals aged ≤ 40 years, ranging from 6.5% (95% CI 5.6–7.5%) to 9.1% (95% CI 8.9–9.3%) reported in previous studies.^[32,41]^ Similarly, the reported prevalence rates of ACN ranging from 0.86% (95% CI 0.8–0.93%) to 1.1% (95% CI 0.8–1.6%) were also lower than our rate.^[32,41]^ These findings support the recommendations regarding the starting age of CRC screening in individuals with FDRs diagnosed with CAA, regardless of whether these individuals were under the age of 40 years. Upon stratifying according to the sex of siblings, we observed that the prevalence of CAA was significantly higher in male siblings than in their female counterparts. Recent studies conducted in Vietnam have indicated a higher prevalence of CAA in males compared to females.^[42,43]^ In a global context, the findings from 2 additional large reviews indicated a greater prevalence of CAA among males compared to females.^[25,34]^ However, the generalizability of these findings to other populations may be limited by the study’s single-center design and its focus on siblings of probands with advanced-adenoma. Further multicenter studies across diverse populations are needed to confirm this association and assess its global relevance. The prevalence of colorectal neoplasia and ACN among male siblings were 28.3% and 15.2%, respectively. The prevalence of colorectal neoplasia among female siblings was 22%, and none of them were diagnosed with ACN. These findings suggest that CRC screening should be prioritized for male siblings of individuals diagnosed with E-CAA.

There are inherent limitations to our study. First, individuals without a family history were not recruited for direct comparisons with siblings of patients diagnosed with CAA. However, by comparing with the current literature, we found that the prevalence of all colorectal neoplasia and CAA in siblings of patients diagnosed with E-CAA surpassed that in individuals aged ≤ 50 years. In addition, the siblings included in our study were selected according to the age criteria recommended by established guidelines. Such an approach may facilitate a more precise estimation of CAA prevalence in siblings of E-CAA patients. Second, as a single-center study, with 79% (580/732) of identified siblings aged between 18 and 50 years old declining participation, the generalizability of the findings may be limited. However, this observation aligns with previous reports of low compliance with colonoscopic screening among FDRs of patients with large adenomas.^[44]^ This emphasizes the barriers faced in persuading individuals with FDRs affected by CAA, particularly those under 50 years, to undertake colonoscopy. Potential reasons for declination, inferred from the literature, may include fear or discomfort associated with colonoscopy, lack of perceived urgency due to absence of symptoms, logistical barriers such as time or travel constraints, or limited awareness of familial risk.^[5,44]^ These factors underscore the need for targeted educational interventions and streamlined colonoscopy pathways to improve uptake in this high-risk group. Future studies should systematically collect data on reasons for refusal to better address these barriers and improve participation rates.

In conclusion, colorectal neoplasia and ACN, especially adenomas and CAA, are not uncommon in siblings aged ≤ 50 years of E-CAA patients. This prevalence was more pronounced among male siblings. These findings underscore the importance of early CRC screening for high-risk individuals. Additional studies are needed to comprehensively assess the risk of CAA in siblings aged ≤ 50 years of E-CAA patients compared to individuals lacking a family history.

## Acknowledgments

We are grateful to Drs. Truc Le-Thanh Tran, Mai Ngoc Luu, Quang Dinh Le, Doan Thi-Nha Nguyen, Nhu Thi-Hanh Vu, Uyen Pham-Phuong Vo, and Vy Ly-Thao Tran for data assistance at the University of Medicine and Pharmacy at Ho Chi Minh City and the University Medical Center, Ho Chi Minh City.

## Author contributions

**Conceptualization:** Duc Trong Quach.

**Data curation:** Luan Minh Dang, Nhan Quang Le, Huy Minh Le, Diem Thi-Ngoc Vo, Minh Cuong Duong, Duc Trong Quach.

**Formal analysis:** Nguyen Lam Vuong, Duc Trong Quach.

**Methodology:** Luan Minh Dang, Duc Trong Quach.

**Supervision:** Luan Minh Dang, Nhan Quang Le, Duc Trong Quach.

**Writing – original draft:** Luan Minh Dang, Minh Cuong Duong, Duc Trong Quach.

**Writing – review & editing:** Luan Minh Dang, Nhan Quang Le, Huy Minh Le, Diem Thi-Ngoc Vo, Nguyen Lam Vuong, Minh Cuong Duong, Duc Trong Quach.
